# Value of the Ratio of Monocytes to Lymphocytes for Monitoring Tuberculosis Therapy

**DOI:** 10.1155/2019/3270393

**Published:** 2019-05-27

**Authors:** Wei Wang, Li-fei Wang, Yan-yan Liu, Fang Yang, Lei Zhu, Xian-hui Zhang

**Affiliations:** ^1^Department of Laboratory Medicine, Shanxi Provincial People's Hospital, Taiyuan, China; ^2^Department of Tuberculosis, The Fifth People's Hospital of Taiyuan, Taiyuan, China; ^3^Department of Laboratory Medicine, Children's Hospital of Shanxi Province, Taiyuan, China

## Abstract

**Objective:**

The objective of this study was to evaluate the change of the ratio of monocytes to lymphocytes in patients with active tuberculosis, such as to provide reference for clinical diagnosis and treatment.

**Methods:**

All data were collected from the clinical database of The Fifth People's Hospital of Taiyuan, China. A total of 151 patients who had newly diagnosed active tuberculosis with tuberculosis therapy in hospital and 129 healthy controls were selected.

**Results:**

Median ratio of monocytes to lymphocytes was 0.45 (IQR: 0.28–0.67) in patients before treatment and 0.32 (IQR: 0.25–0.46) on discharge (*P* < 0.001).

**Conclusions:**

Ratio of monocytes to lymphocytes may be applied in diagnosis and the chemotherapeutic efficacy of active tuberculosis.

## 1. Introduction

Tuberculosis (TB) is a chronic respiratory infectious disease caused by *Mycobacterium tuberculosis* affecting one-third of the world's population, ten percent of whom developed active tuberculosis [1, 2]. Although the morbidity and mortality has declined obviously, but along with the increase in drug-resistant strains of the TB bacterium, its treatment difficulty is also growing [3]. The immune system status plays an important role in tuberculosis infection. Monocytes cells have been considered as the target cells of *Mycobacterium tuberculosis*, and lymphocytes are the main effector cells of TB immunity [4]. As the key immune cells, the levels of monocyte and lymphocyte might reflect the state of an individual's immune to infection. Full blood count of peripheral blood is the most frequently performed test in clinical practice, but the ratio of monocyte to lymphocyte (MLR), as a simple biomarker, is not commonly used in clinical care.

The association between MLR and risk of mycobacterial infections has been first reported in rabbits in the 1920s by Florence Sabin and colleagues [5, 6]. They reported that the MLR in peripheral blood might reflect the extent and progress of the TB disease in rabbit models. Recent published studies suggest that the elevated MLR may be associated with the risk of active tuberculosis in adults, infants, and postpartum women with HIV infection [7, 8]. In this study, we evaluate the peripheral blood levels of MLR in active TB patients to try to determine whether the MLR will be affected by TB or TB treatment and offer a new reference for clinical diagnosis and treatment outcome.

## 2. Materials and Methods

### 2.1. Study Participants

A total of 151 patients with active TB and 129 healthy controls were included into the study. Patients combined with other pathogen infection were excluded. All patients' information was collected from the clinical data of The Fifth People's Hospital of Taiyuan between March 1, 2017, and September 1, 2018. Healthy controls were selected from people of physical examination in Shanxi Provincial People's Hospital. The study was approved by the ethical committee of the Fifth People's Hospital of Taiyuan.

All inpatients were diagnosed and treated in accordance with Chinese Guidelines on the Diagnosis and Treatment of tuberculosis. Discharge criteria includes the following: negative sputum smear for acid-fast bacilli, negative culture for sputum mycobacterium, chest X-rays improved, absorption of pleural effusion, the erythrocyte sedimentation depressing, or enlarged lymph nodes shrinking.

### 2.2. Full Blood Counts

EDTA blood specimens of all patients were collected at the first day of admission and discharge. Leukocyte differential counts were performed by standard procedures on a Sysmex automated hematology analyzer. The MLR was calculated as the quotient of the absolute monocyte and lymphocyte counts.

## 3. Statistical Analysis

All quantitative data were presented using median and interquartile range. A nonparametric Wilcoxon matched pairs test was used to compare the variables before treatment and after treatment in TB patients. The Mann–Whitney *U* test was used to compare the variables between patients and controls. Two-sided *P* value of less than 0.05 was considered as statistical significance. The statistical package for IBM social science (SPSS) version 22.0 and GraphPad Prism 5 software was used to analyze all data.

## 4. Results

### 4.1. Demographic Description of the Study Subjects

A total of 151 patients with active TB were included into the study. They were all inpatients diagnosed with pulmonary TB, intestinal TB, pelvic TB, TB peritonitis, and TB pleurisy from the Fifth People's Hospital of Taiyuan between March 1, 2017, and September 1, 2018. The mean age of the 151 patients was 36 ± 16.58 years, and 91 (60%) were male. The average hospitalization time was 39.93 ± 13.19 days. The healthy control group consisted of 75 males and 54 females, with an overall mean age of 38 ± 11.7 years. There was no significant difference in age (*P*=0.11) and gender (*P*=0.72) between the patients and the healthy control group.

### 4.2. Peripheral WBC Counts in Healthy Controls and TB Patients before Treatment and on Discharge

**Figure 1:**
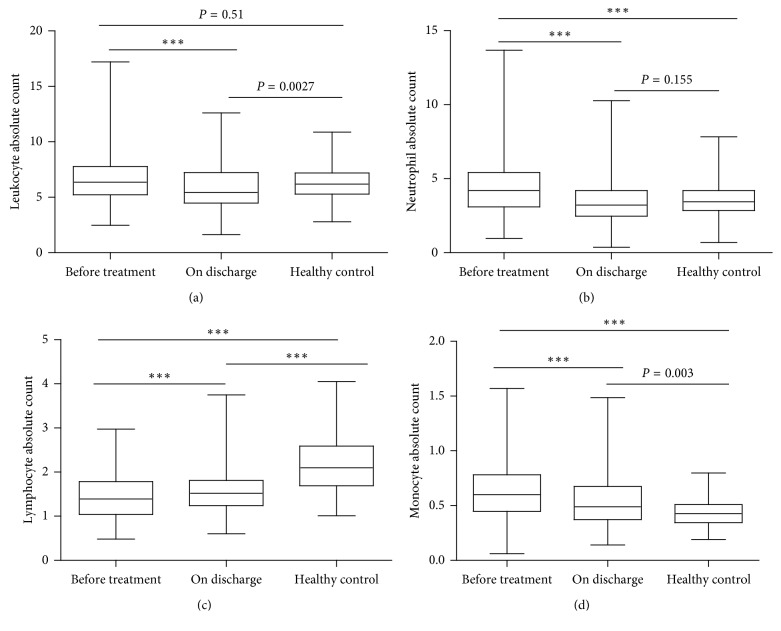
Comparison of the WBC absolute count in patients and healthy control: (a) leukocytes; (b) neutrophils; (c) lymphocytes; (d) monocytes. Data are presented with the boxplot graph reporting the median and the interquartile rang (^*∗∗∗*^*P* < 0.001).

We measured peripheral WBC counts in TB patients before treatment and on discharge to assess the variation of WBC subpopulations. As shown in Figure 1(a), the leukocyte count reduced significantly in TB patients on discharge when compared to the count before treatment. Similarly, the neutrophil and monocyte counts were significantly decreased after treatment (Figures 1(b) and 1(d)). Meanwhile, the increased lymphocyte count on discharge was also highly significant (Figure 1(c)). Compared to the healthy controls, there was a trend that the WBC subpopulations were moving towards normal with successful treatment in TB patients.

### 4.3. Association between MLR and TB Disease

We calculated the ratio of monocytes to lymphocytes to evaluate the impact of active TB disease and anti-TB therapy on the MLR. The MLR of patients before treatment was 0.45 (IQR: 0.28–0.67), which was higher significantly than healthy controls (0.20, IQR: 0.17–0.25) (*P* < 0.001). After anti-TB treatment, the MLR of patients on discharge was 0.32 (IQR: 0.25–0.46), which was decreased significantly and still higher than healthy controls (*P* < 0.001).

### 4.4. Change of MLR with Anti-TB Treatment in Different Subgroups

We divided patients into different groups by gender, age, and TB location to assess the change of MLR between before treatment and on discharge. As shown in Table 1, there were highly significant differences in MLR of patients between before treatment and on discharge in different groups (Table 1).

## 5. Discussion

Interactions between mycobacterium and host immune system resulted in the outcome of tuberculosis [9]. Monocytes/macrophages are derived from hematopoietic stem cells in the bone marrow and circulate in the bloodstream. These cells play a determinant role in innate immune response and act as a link to the adaptive immune response due to their antigen presenting function. In the progression of TB infection, monocytes/macrophages can phagocytize and restrict mycobacterium and be recruited to form granuloma [10]. The increase of MLR indicated the relative increase of monocytes and the relative decrease of lymphocytes. The ratio may reflect the efficiency of immune response against infection. Studies have shown that an elevated percentage of monocytes in peripheral blood and an increased MLR are associated with the risk of tuberculosis [11]. Besides, the MLR seems to be a prognostic factor in patients with various diseases, such as various cancers, diabetes, and cardiovascular disease [8, 12–17].

Naranbhai et al. found that MLR could predict the risk of developing tuberculosis during follow-up by analyzing the peripheral blood cells of 1,336 South African infants aged 3-4 months [8]. They also indicated that monocyte functional and transcriptional differences are dependent on the MLR which suggests that qualitative differences in monocytes are better reflected by the MLR than by monocyte counts alone [4]. TB infection disturbed the normal MLR by activating immune cells in peripheral blood. It has been also reported that TB infection may alter subsets of the hematopoietic stem cell to change the peripheral blood counts [18]. The detailed mechanisms are required to be further elucidated. The variations of WBC subpopulations in active TB have been further demonstrated in our study. There was an elevated neutrophil and monocyte count and a reduced lymphocyte count in active TB patients compared to the healthy controls. The MLR may reflect the relative frequency of monocytes as target cells and lymphocytes as effectors against TB [8]. Our study showed that active TB patients before treatment had a significant higher MLR than healthy controls, which is consistent with the previous studies [11, 19].

In order to evaluate the effect of anti-TB therapy on MLR, we analyzed the difference between before treatment and on discharge. We found that the MLR of patients on discharge decreased significantly, which suggested that the MLR may be changed with anti-TB therapy. At the same time, the differences were analyzed in different ages, genders, and TB location, respectively. The results showed there was statistically significant difference in all groups, which indicated that the factors did not influence the change of MLR. Wang et al. [20] analyzed the MLR changes before and after treatment of 108 active TB patients with completed therapy and indicated that the MLR returned to normal after treatment [16]. The other study also found there was no difference between the MLR of cured TB and healthy donors [19]. However, our results showed that the MLR of patients after treatment was still higher than healthy controls. That is because our data were collected on discharge, and the anti-TB therapy was not completed. Nevertheless, the study showed the trend that the MLR of patients recovered to normal with treatment. This change suggested that the MLR may reflect the effectiveness of anti-TB therapy.

Current indicators to evaluate the efficacy of TB therapy have sputum smear with acid-fast bacilli turning negative, the sputum *Mycobacterium* cultivation overcast, the absorption of pleural and ascetic fluid, pulmonary shadows subsiding, calcification, fibrosis, and erythrocyte sedimentation depressing. However, the indicators cannot be used to evaluate the TB treatment of pelvic tuberculosis patients and patients with drug-resistant TB early treatment. Moreover, the indicators need several days to get results. In order to ensure disease control, treatment for an extend period of time was required. This study suggests that MLR may be as a simple biomarker for evaluating the efficacy of pelvic tuberculosis and early treatment of drug-resistant tuberculosis.

In present, there are little evidence about the MLR and tuberculosis. Our research as a retrospective study has several limitations. Firstly, the sample size was small. Secondly, we only collected the data of peripheral blood at the time of discharge. The MLR after treatment should be collected to forecast the long-term prognosis. Besides, the MLR may have a value in early detection of treatment. The MLR should be collected in different times of treatment to make the study more valuable. Thirdly, the MLR is a nonspecific biomarker which is affected by the combination of inflammation, immune system diseases, and other tumors. The MLR of active TB patients should be compared with other respiratory disease patients to provide more information. Further studies should endeavor to assess more detailed information.

To sum up, our study showed that the MLR, as a simple, convenience, and low-cost marker, may be used for diagnosis and evaluates the response of therapy in active TB. However, more large-scale and prospective studies are needed to confirm the reliability of this study. Of course, the test cannot replace clinical symptoms and traditional examinations and should be used together to get more perfect results.

## Figures and Tables

**Table 1:** MLR of patients before treatment and on discharge in different subgroups.

	*N*	Before treatment	On discharge	*P*
Gender				
Male	90	0.50 (0.32–0.69)	0.35 (0.26–0.54)	<0.001
Female	61	0.37 (0.24–0.62)	0.30 (0.20–0.39)	<0.001
Age				<0.001
<30 y	75	0.47 (0.28–0.69)	0.32 (0.26–0.46)	<0.001
30–55 y	46	0.38 (0.25–0.61)	0.29 (0.21–0.41)	<0.001
>55 y	30	0.47 (0.32–0.77)	0.35 (0.23–0.59)	<0.001
Location				<0.001
Pulmonary TB	120	0.44 (0.26–0.45)	0.32 (0.25–0.45)	<0.001
Extrapulmonary TB	31	0.49 (0.27–0.77)	0.37 (0.24–0.48)	0.001

## Data Availability

The datasets used or analyzed during the current study are available from the corresponding author on reasonable request.
